# Effects of *Clostridium butyricum* on growth performance, metabonomics and intestinal microbial differences of weaned piglets

**DOI:** 10.1186/s12866-021-02143-z

**Published:** 2021-03-22

**Authors:** Jing Liang, Shasha Kou, Cheng Chen, Sayed Haidar Abbas Raza, Sihu Wang, Xi Ma, Wen-Ju Zhang, Cunxi Nie

**Affiliations:** 1 College of Animal Science and Technology, Shihezi University, Shihezi, Xinjiang, 832003 People’s Republic of China; 2 College of Animal Science and Technology, Northwest A&F University, Yangling, Shaanxi 712100 People’s Republic of China; 3 State Key Laboratory of Animal Nutrition, College of Animal Science and Technology, China Agricultural University, Beijing, 100193 People’s Republic of China

**Keywords:** *Clostridium butyricum*, Growth performance, Intestinal microbiota, Metabolomics, Weaned piglet

## Abstract

**Background:**

Weaning stress of piglets causes a huge economic loss to the pig industry. Balance and stability of the intestinal microenvironment is an effective way to reduce the occurance of stress during the weaning process. *Clostridium butyricum*, as a new microecological preparation, is resistant to high temperature, acid, bile salts and some antibiotics. The aim of present study is to investigate the effects of *C. butyricum* on the intestinal microbiota and their metabolites in weaned piglets.

**Results:**

There was no statistical significance in the growth performance and the incidence of diarrhoea among the weaned piglets treated with *C. butyricum* during 0–21 days experimental period. Analysis of 16S rRNA gene sequencing results showed that the operational taxonomic units (OTUs), abundance-based coverage estimator (ACE) and Chao index of the CB group were found to be significantly increased compared with the NC group (*P* < 0.05). Bacteroidetes, Firmicutes and Tenericutes were the predominant bacterial phyla in the weaned piglets. A marked increase in the relative abundance of *Megasphaera*, *Ruminococcaceae*_NK4A214_group and *Prevotellaceae*_UCG-003, along with a decreased relative abundance of *Ruminococcaceae*_UCG-005 was observed in the CB group, when compared with the NC group (*P* < 0.05). With the addition of *C. butyricum*, a total of twenty-two significantly altered metabolites were obtained in the feces of piglets. The integrated pathway analysis by MetaboAnalyst indicated that arginine and proline metabolism; valine, leucine and isoleucine biosynthesis; and phenylalanine metabolism were the main three altered pathways, based on the topology. Furthermore, Spearman’s analysis revealed some altered gut microbiota genus such as *Oscillospira*, *Ruminococcaceae*_NK4A214_group, *Megasphaera*, *Ruminococcaceae*_UCG-005, *Prevotella*_2, *Ruminococcaceae*_UCG-002, *Rikenellaceae*_RC9_gut_group and *Prevotellaceae*_UCG-003 were associated with the alterations in the fecal metabolites (*P* < 0.05), indicating that *C. butyricum* presented a potential protective impact through gut microbiota. The intestinal metabolites changed by *C. butyricum* mainly involved the variation of citrulline, dicarboxylic acids, branched-chain amino acid and tryptophan metabolic pathways.

**Conclusions:**

Overall, this study strengthens the idea that the dietary *C. butyricum* treatment can significantly alter the intestinal microbiota and metabolite profiles of the weaned piglets, and *C. butyricum* can offer potential benefits for the gut health.

## Background

Weaning stressors are generally associated with the changes in environment, diet, management, and separation from the mother that exhibit a negative impact on the growth performance and intestinal health of the young animals, leading to diarrhoea, slow growth, and decreased ability to resist disease [1]. Alleviating stress damage caused by weaning in piglets is an urgent problem to be solved. In the last few years, antibiotics have acted a pivotal part in controlling the diseases in animals, and improving animal growth and reproductive performance. However, antibiotics can improve the tolerance of pathogenic bacteria, kill beneficial bacteria, and may cause a damage to the environment. With this background, the Ministry of Agriculture and Rural Affairs of the People’s Republic of China in 2020 has restricted the use of antibiotics as growth promoters in animal feed. Thus, there is an urgency to find an efficient alternative to reduce the economic losses caused by this prohibition. Several studies have demonstrated that the microbial ecological agents, organic acids, essential oils and probiotics can act as the potential alternatives for the use of antibiotics [2–5].

The probiotic *C. butyricum* is well known gram-positive and obligate anaerobic bacillus [6] that can colonise the intestinal tract of animals [7]. As an important constituent of probiotics, *C. butyricum* has been used to prevent or treat the intestinal disorders of animals [8–10]. Apart from the beneficial effects on intestinal tract, *C. butyricum* addition was recently reported to promote the growth in animals [11–15], improve immune response [16, 17], and regulate the structure and composition of gut microbiota in livestock [18–20]. Gut microbiota affects the digestion and absorption of nutrients [21, 22], and the microbial metabolites associated with them are known to cause majority of the biological effects [23].

Metabolomics has an enormous potential for studying the functions in complex systems and expanding knowledge about gut microbial metabolism [24]. Metabolomics has been used to study the metabolism of pigs at present [25, 26]. Although there are several studies on *C. butyricum* in livestock, the microbiome-metabolomics analysis has been not extensively studied, especially in the piglets. Recent studies have described the microbial production and bioavailabilities of xenometabolites and their derivatives, which makes it important to understand the influence of the microbiota on the host physiology [27]. Therefore, the present study was conducted in order to assess the effects of *C. butyricum* on the growth performance, metabonomics and intestinal microbial differences in weaned piglets by sequencing of the 16S rRNA gene and a liquid chromatography-mass spectrometry (LC-MS) platform based metabolomics. Furthermore, we attempted to explore the possible relationship between the bodyweight, incidence of diarrhea, intestinal microbiota and metabolomics.

## Methods

### Animals, diet, and sampling

Ninety crossbred [(Yorkshire×Landrace) × Duroc] piglets (male: female was 45: 45) with (6.22 ± 0.24) kg of body weight weaned at (28 ± 2) days of age were randomly allocated into 3 groups with 6 replicates per group and 5 piglets in each replicate. They were NC group (control diet, negative control), PC group [control diet + 0.1 g antibiotic (colistin sulphate)/kg of control diet] and CB group (control diet + 5 × 10^8^ CFU *C. butyricum*/kg of control diet). The diets were fed in amounts that met or exceeded National Research Council (NRC) nutrient recommendations [28]. The *C. butyricum* culture was obtained from the Zhejiang Huijia Bio-technology Co., Ltd. (Zhejiang, China). It was isolated from the contents of pig intestines. The strain number was HJCB998 and preserved by China General Microbiological Culture Collection Center (CGMCC). Colistin sulphate was purchased by the veterinary drug factory of Shihezi city. The composition and nutrient content of the control diets are shown in Table 1. The feeding management of piglets, the measurement and calculation of average daily gain (ADG), average daily feed intake (ADFI), feed to gain ratio (F/G) and diarrhoea incidence were followed by our previous study [29]. Faecal samples of the piglets were collected by rectal stimulation on day 21. Three piglets were randomly selected from each group, the faecal samples were stored in cryosurgery tubes, immediately deep-frozen in liquid nitrogen and stored at − 80 °C until further analysis.

**Table 1:** Composition and nutrient levels of control diet (air-dried basis, %)

Ingredients	Content	Nutrient levels^b^	Content
Corn	58.60	Digestible energy (MJ/kg)	13.68
Soybean meal	17.50		
Expanded soybean	7.50	Crude protein	20.40
Milk powder	4.00	Lys	1.30
Fish meal	4.00	Met+Cys	0.78
Whey power	4.30	Thr	0.87
NaCl	0.30	Na	0.27
Limestone	1.22	Ca	0.95
CaHPO_4_	1.16	Available P	0.39
DL-met	0.07		
Lys·HCL	0.35		
Premix^a^	1.00		
Total	100.00		

^a^ The premix provided the per kg of diet as follows: VA 8000 IU, VB_1_ 4 mg, VB_2_ 3.6 mg, VB_5_ 40 mg, VB_6_ 4 mg, VB_12_ 0.02 mg, VD_3_ 3000 IU, VE 20 IU, VK_3_ 2 mg, biotin 0.15, folic acid 1 mg, D-pantothenic acid 11 mg, nicotinic acid 10 mg, antioxidant 100 mg, Cu (as copper sulfate) 10 mg, Fe (as ferrous sulfate) 80 mg, Mn (as manganese sulfate) 30 mg, Zn (as zinc sulfate) 75 mg, I (as potassium iodide) 0.4 mg, Se (as sodium selenite) 0.3 mg. ^b^ Digestible energy and Available P were calculated values, while the others were measured values

### DNA extraction, illumina miseqsequencing, and data processing

Microbial DNA was extracted from the faecal samples utilizing the TIANamp Stool QIAamp PowerFecal DNA Kit (QIAGEN, Hilden, Germany) according to the manufacturer’s protocols [26]. DNA concentration and purification were settled determined by NanoDrop 2000 UV-vis spectrophotometer (Thermo Scientific, Wilmington, USA), and the DNA quality was checked by 1% agarose gel electrophoresis. The hypervariable regions of the bacteria 16S rRNA gene were amplified with primers 515F (5′-GTGCCAGCMGCCGCGGTAA-3′) and 907R (5′-CCGTCAATTCCTTTGAGTTT-3′) [30] by utilizing a thermocycler PCR system (GeneAmp 9700, ABI, USA). PCR cycles were conducted using an accompanying programme: denaturation of 3 min at 95 °C, subjected to 27 cycles of 30 s at 95 °C, 30 s for annealing at 55 °C, elongation of 45 s at 72 °C, followed by a final extension step for 10 min at 72 °C. PCR were performed in triplicate 20 μL mixtures including 4 μL of 5 × FastPfu Buffer, 2 μL of 2.5 mM dNTPs, 0.8 μL of each primer (5 μM), 0.4 μL of FastPfu Polymerase and 10 ng of template DNA. The resultant PCR products were collected from a 2% agarose gel and then further purified with an AxyPrep DNA Gel Extraction Kit (Axygen Biosciences, Union City, CA, USA) and quantified by using QuantiFluor™-ST (Promega, USA) according to the manufacturer’s suggested protocol. Purified amplicons were pooled in equimolar solutions and comprised 2 × 300-bp paired-end reads on an Illumina MiSeq platform (Illumina, San Diego, USA) [9] according to the standard protocols by Majorbio Bio-Pharm Technology Co. Ltd. (Shanghai, China). The raw sequencing data were submitted to the NCBI Sequence Read Archive (SRA, NCBI, http://www.ncbi.nlm.nih.gov/sra) as available under accession number PRJNA679135.

Raw FASTQ files were quality-filtered by Trimmomatic and merged by Fast Length Adjustment of Short Reads (FLASH) based on the following criteria: The reads were truncated at any site receiving an average quality score < 20 over a 50 bp sliding window. Sequences whose overlap was longer than 10 bp were merged according to their overlap with mismatch, not more than 2 bp. Sequences of each sample were separated, primers, read and the ambiguous bases were removed [31, 32]. OTUs were obtained based on a 97% similarity cut-off by UPARSE (version 7.1 http://drive5.com/uparse/) [9]. The taxonomy of each sequence of 16S rRNA gene was analysed by the Ribosomal Database Project (RDP) Classifier algorithm (http://rdp.cme.msu.edu/) against the full SILVA 16S rRNA gene reference database (https://www.arb-silva.de/) with a 70% confidence threshold [9].

### LC-MS analysis

The chromatographic separation was carried out on an ACQUITY UPLC® HSS T3 column maintained at 40 °C using an ACQUITY UPLC system. The column dimensions and particle size were 2.1 × 150 mm, 1.8 μm. The autosampler temperature was set at 4 °C. Gradient elution of the analytes was carried out with a mobile phase of 0.1% (v/v) formic acid in water (A) and 0.1% (v/v) formic acid in acetonitrile (B). The flow rate for the mobile phase was 0.25 mL/min. The injection volume was 6 μL for each sample after equilibration. A linear gradient was run as follows: 0–1 min, maintain at 2% solvent B; 1–9.5 min, 2–50% solvent B; 9.5–14 min, 50–98% solvent B; 14–15 min, 98% solvent B; 15–15.5 min, 98–2% solvent B; and 15.5–17 min, 2% solvent B [33].

The ESI-MSn experiments were performed on a Thermo LTQ-Orbitrap XL mass spectrometer (Bremen, Germany) with the spray voltage in positive ion mode was 4.8 kV and 4.5 kV in negative ion mode. Auxiliary gas and sheath gas were maintained at 15 and 45 arbitrary units and capillary temperature was set at 325 °C. The voltages of the tube and capillary were 50 V and 35 V in positive mode, 50 V and 15 V in negative mode. An overall mass range of 50–1000 m/z was scanned by the Orbitrap analyser and the full scan has reached a resolution of 60,000. Data-dependent acquisition (DDA) MS/MS experiments were executed with a collisionally induced dissociation (CID) scan at 30 eV collision energy. With dynamic exclusion enabled. Dynamic exclusion was set to a repeat count of 2 and an exclusion duration was carried out 15 s [34].

The raw files were changed to the MzXML format (XCMS input file format) using the ProteoWizard software (V3.0.8789) [35]. The XCMS package of R (v3.3.2) was used for the detection of peaks, peak filtration and peak alignment [36]. Then, a two-dimensional data matrix, consisting of the mass to charge ratio (m/z), retention time (RT) and peak area of metabolites was obtained and introduced into SIMCA-P 13.0 software (Umetrics AB, Umea, Sweden) for multivariate statistical analysis [37]. Partial least squares-discriminate analysis (PLS-DA) was carried out to predict, describe modelling and discriminate variable selection [38]. Both the variable importance under the fold change (FC) values (FC≧1.5 or FC≦0.667) obtained from the OPLS-DA and t-test (*P* < 0.05) were engaged to analyse the differentially expressed metabolites between the two comparison groups [39]. Then, the precise MS/MS fragmentation information was inquired by the Human Metabolome Database (HMDB, http://www.hmdb.ca/), the Metlin database (http://metlin.scripps.edu/), KEGG (http://www.genome.jp/kegg/) for screening and identification of potential biomarker [40, 41]. The metabolic pathways were analyzed through MetaboAnalystR 3.0 (http://www.metaboanalyst.ca).

### Data analysis

Data on growth parameters and diarrhoea incidence were collected and analyzed using one-way analysis of variance (ANOVA) followed by Duncan’s multiple range tests. Alpha diversity indices data was presented as “mean ± standard deviation (SD)”, and analysed by ANOVA with least significant difference (LSD) test. Employing MOTHUR (version v.1.30.1 http://www.mothur.org/) to ascertain bacterial community composition and structure. Heat map was obtained using the R packages VEGAN (version 3.1.0, http://www.microbesonline.org/fasttree/) [42]. Non-metric multidimensional scaling (NMDS) analysis was conducted using the VEGAN package of R (V3.1.0) software (http://www.r-project.org/). The community histograms and Venn diagrams were drawn using Origin 8.0 (OriginLab, Northampton, MA, USA). The connection between the significantly changed bacteria at the genus level and metabolites (FC≦0.667 or FC≧1.5, adjusted *P* < 0.05) were evaluated by Spearman’s rho tests. Correlation maps between the body weight, diarrhea incidence, differential microbiota and metabolites were attained from the cloud platform of Gene denovo (https://www.omicshare.com/website). Statistical calculations in the present study were based on Version 22.0 of SPSS for Windows by SPSS Inc. and a *P* value < 0.05 was accepted as statistically significant.

## Results

### Growth performance

As is shown by the data in Table 2 that no significant difference was found in the ADG, ADFI and F/G among the three treatments during each period (*P* > 0.05). One interesting finding was that diarrhoea incidence was significantly greater in the PC and CB group than NC group during the 14–21 day feeding (*P* < 0.05). No difference in the diarrhoea incidence was noted during the intervals of 0–7 day, 7–14 day and 0–21 day (*P* > 0.05). **(Table** **2****).**

**Table 2:** Effects of *C. butyricum* on growth performance and diarrhoea incidence in weaned piglets

	Treatments^b^				
Item^a^	NC	PC	CB	SEM^3^	*P*-value
Initial weight (kg)	6.19	6.09	6.38	0.08	0.352
Final weight (kg)	13.08	13.13	13.40	0.23	0.862
d 0–7					
ADG(g)	151.36	171.09	161.22	5.36	0.370
ADFI(g)	232.65	232.65	226.87	1.41	0.150
F/G	1.54	1.38	1.41	0.05	0.361
Diarrhea incidence (%)	10.20	9.52	6.12.	1.95	0.717
d 7–14					
ADG(g)	360.20	313.61	325.85	9.73	0.115
ADFI(g)	380.27	382.31	358.16	8.86	0.531
F/G	1.07	1.22	1.10	0.04	0.342
Diarrhea incidence (%)	15.99	9.52	2.38	3.84	0.403
d 14–21					
ADG(g)	472.45	521.43	516.33	10.76	0.109
ADFI(g)	580.27	581.97	567.69	5.87	0.620
F/G	1.23	1.12	1.10	0.03	0.125
Diarrhea incidence (%)	4.08^b^	11.22^a^	9.86^a^	1.37	0.047
d 0–21					
ADG(g)	328.00	335.37	334.47	10.05	0.961
ADFI(g)	397.73	398.98	384.24	4.96	0.463
F/G	1.22	1.20	1.18	0.04	0.930
Diarrhea incidence (%)	10.09	10.09	6.12	1.85	0.663

^a^ADG, average daily gain; ADFI, average daily feed intake; F/G, feed to gain ratio. ^b^Piglets were fed different diets. NC, negative control, control diet; PC, positive control, control diet + 0.1 g/kg antibiotic (colistin sulphate); CB, control diet + 5 × 10^8^ CFU/kg of *C. butyricum*. ^3^SEM, total standard error of means. (*n* = 6)

### Bacterial community structure

A amount of 1,225,756 paired-end reads, including 307,664,756 bp were produced by the raw data, and 368,348 valid sequences remained after chimaeras were filtered out and low-quality sequences were eradicated. Microbial diversity indexes are displayed in Table 3. A total of 463 OTUs were identified from all groups. The coverage of the three treatments was above 99%, demonstrating that the sequencing reads were sufficient for this analysis. OTUs and statistical estimates of the species diversity [25] and richness (ACE, Chao) for each treatment at a genetic distance of 3% are shown in Table 3. A closer inspection of the Table 3 shows that the CB group exhibited a significantly high OTU (*P* = 0.03, *P* = 0.029) and a significantly high index of ACE (*P* = 0.036, *P* = 0.044), in comparison to the NC and PC groups. The Shannon index of the CB group showed an increasing tendency compared to that in the NC and PC groups (*P* = 0.064, *P* = 0.07), but none of these differences was statistically significant. In addition, the CB group had a significantly greater Chao index value than the NC group (*P* = 0.045). Overall, these results indicate that the CB treatment improved the rectal bacterial richness and diversity. **(**Table 3**).**

**Table 3:** Alpha diversity indices of rectal bacterial communities in weaned piglets

		Treatments^a^		
Item	NC	PC	CB	*P*-value
OTUs	293.00 ± 45.53^b^	292.67 ± 49.22^b^	386.00 ± 18.08^a^	0.046
Coverage, %	99.85 ± 0.02	99.82 ± 0.04	99.84 ± 0.02	0.523
Shannon	3.69 ± 0.33	3.70 ± 0.30	4.19 ± 0.13	0.107
ACE	318.21 ± 42.67^b^	323.38 ± 53.71^b^	408.63 ± 19.60^a^	0.062
Chao	321.21 ± 38.30^b^	332.74 ± 59.09^ab^	409.18 ± 22.09^a^	0.087

^a^ Piglets were fed different diets. NC, negative control, control diet; PC, positive control, control diet + 0.1 g/kg antibiotic (colistin sulphate); CB, control diet + 5 × 10^8^ CFU/kg of *C. butyricum*. Values are represented as mean ± SD (*n* = 3), and the variant letter in the same row indicated significant difference when *P* < 0.05

### Specific microbial phyla and genera among different feeding methods

**Fig. 1:**
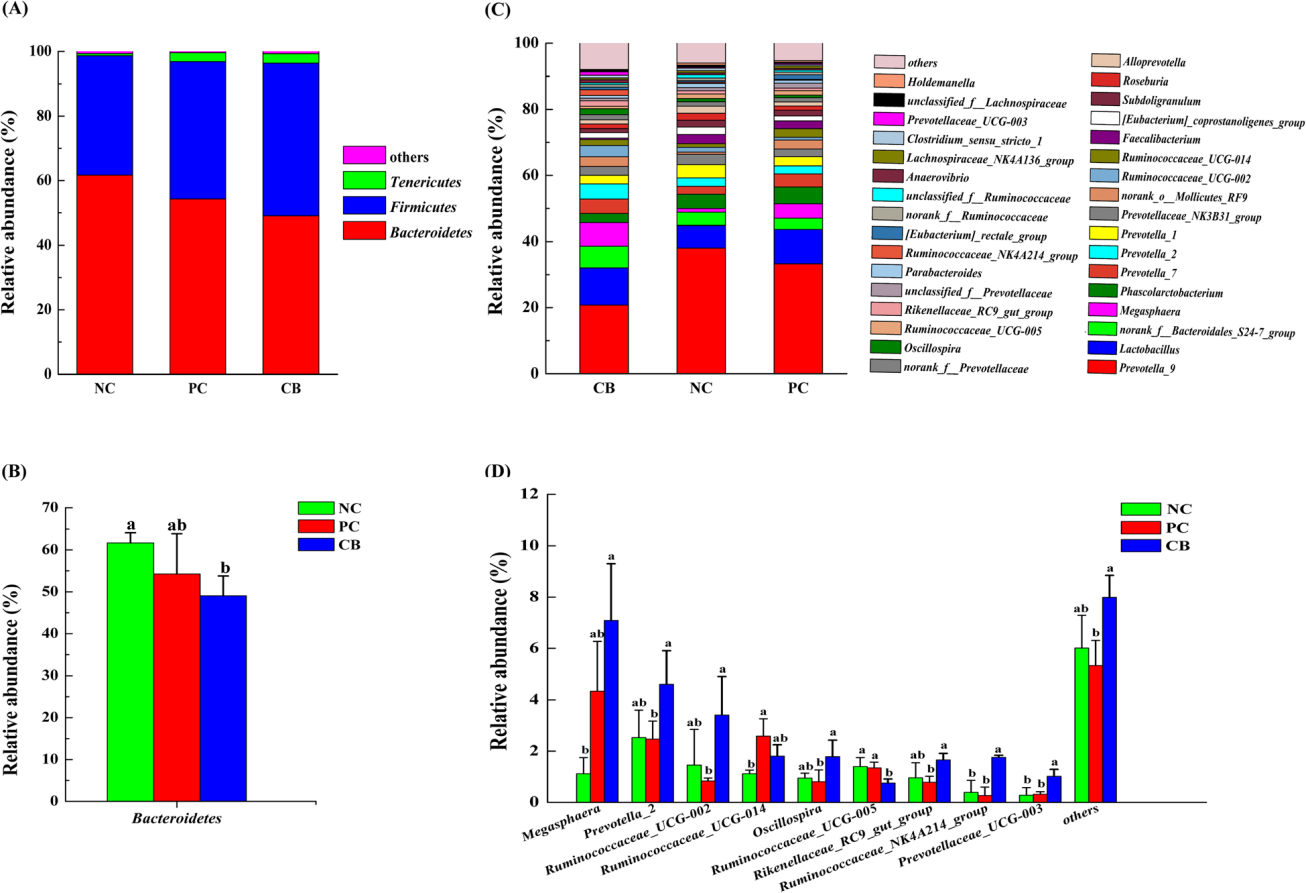
Classification of the bacterial community composition across the three different treatment groups. **a** Relative abundance of bacterial phylum level. **b** Extended error bar plot showing the bacteria at the phylum level that had significant differences among the three groups. **c** Relative abundance of bacterial genus level. **d** Extended error bar plot showing the bacteria at the genus level that had significant differences among the three groups. Data presented as mean ± standard deviation, *n* = 3. Different lowercase indicates significant difference (*P* < 0.05). NC, negative control, control diet, PC, positive control, control diet + 0.1 g/kg antibiotic (colistin sulphate), CB, control diet + 5 × 10^8^ CFU/kg of *C. butyricum*

The bacterial community composition in rectal contents at the phylum and genus level is presented in Fig. 1. There were three phyla with the abundance of ≧0.01% in each group, including Bacteroidetes, Firmicutes, and Tenericutes. Bacteroidetes and Firmicutes accounted for a relative abundance of 48.77 ~ 61.85% and 36.86 ~ 47.62%, respectively, followed by Tenericutes at 0.62 ~ 2.94%. In comparison to the NC group, the abundance of Bacteroidetes was significantly declined in the CB group (*P =* 0.05) **(**Fig. 1**b)**, there was a rising trend of Firmicutes in the CB group (*P =* 0.065). At the genus level, 128 genera were classified and the abundance of 26 of these genera was ≧0.01%. The relative abundance of *Prevotella*_9 in the NC, PC and CB groups was 38.77, 33.51 and 20.42%, respectively. The relative abundance of *Lactobacillus* was 6.80, 10.30, and 11.61%, respectively. The relative abundance of *Bacteroidales*_S24-7_group was 3.99, 3.32, 6.70%, respectively. The relative abundance of *Megasphaera* was 1.11, 4.32, 7.22%, respectively. In contrast to the NC group, the relative abundance of *Prevotella*_9 showed a tendency to decrease in the CB group (*P =* 0.067). Comparing with the NC group, *Megasphaera*, *Ruminococcaceae*_NK4A214_group and *Prevotellaceae*_UCG-003 abundance were substantially increased in the CB group (*P <* 0.05). *Prevotella*_2, *Ruminococcaceae*_UCG-002, *Oscillospira*, *Rikenellaceae*_RC9_gut_group, *Ruminococcaceae*_NK4A214_group and *Prevotellaceae*_UCG-003 abundance were markedly improved in the CB group compared with that in the PC group (*P <* 0.05). Compared with the NC group, the PC group appeared a huge increase in *Ruminococcaceae*_UCG-014 abundance (*P <* 0.05). The relative abundance of *Ruminococcaceae*_UCG-005 in CB group was significantly reduced compared with NC and PC groups (*P <* 0.05) **(**Fig. 1d**).**

**Fig. 2:**
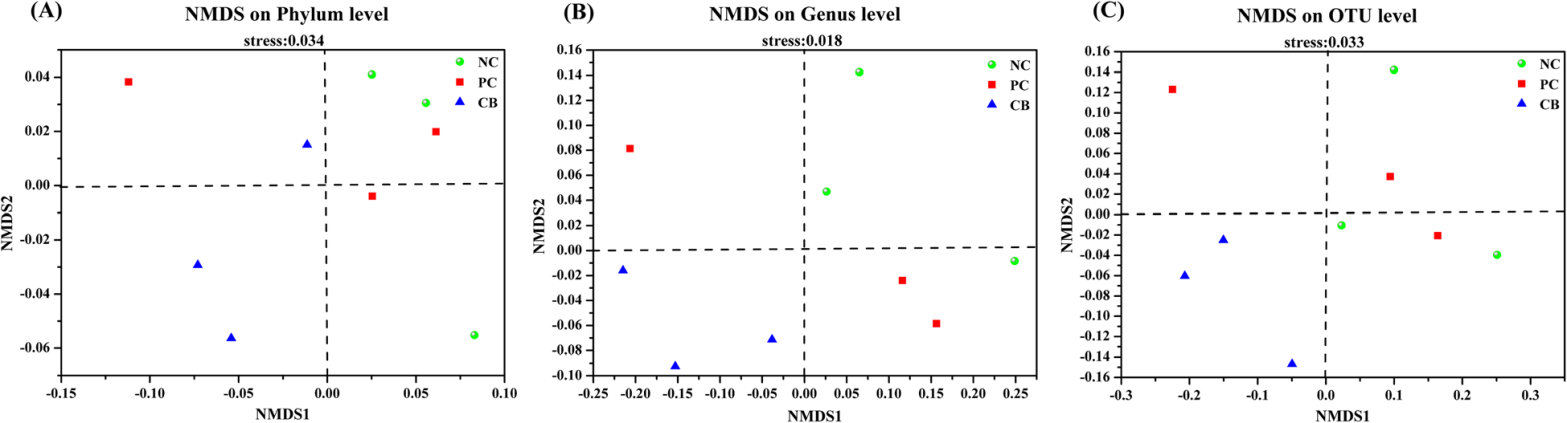
Results from NMDS unconstrained ordinations of samples based on the unweighted Uni Frac distances at phylum, genus and OTU levels. **a** Based on phylum distribution. **b** Based on genus level information. **c** Based on total OTU distribution

The similarity between the bacterial communities of different treatments was confirmed by the NMDS analysis. The stress values of the NMDS differed among the phylum, genus and OTUs. A much better discrimination was observed at the genus and OTUs levels, and samples from the CB treatment clustered together, whereas other groups had no clear boundary (Fig. 2b, c). The results of the analysis of similarities demonstrate a slight descending trend between NC group and CB group, indicating that the bacterial community was sensitive to the addition of *C. butyricum* (R = 0.704, *P* = 0.088) **(**Fig. 2**).**

**Fig. 3:**
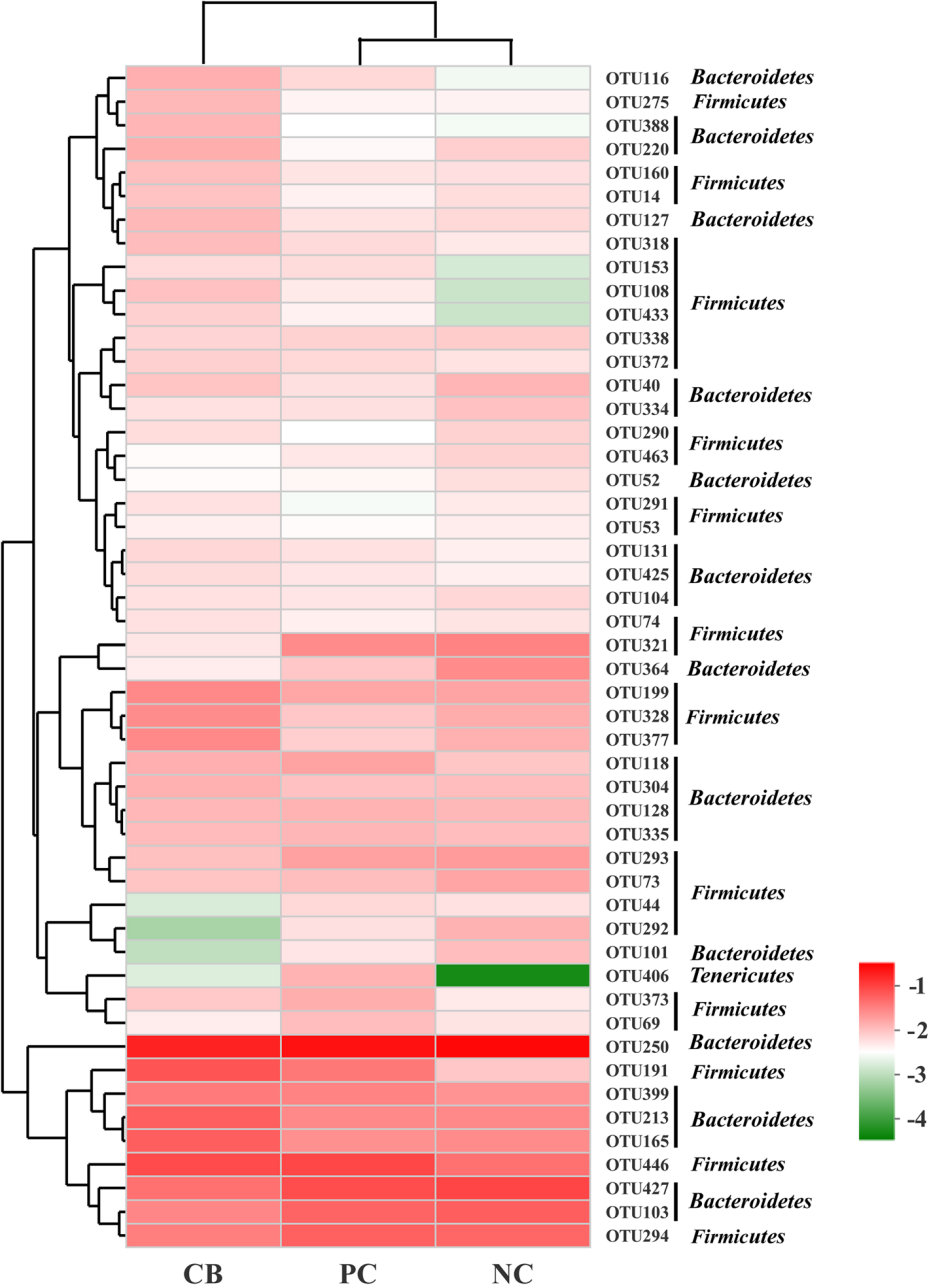
Heatmap showing the most relative abundance of dominant bacterial OTUs. The relative values are indicated by color intensity with the legend indicated at the right corner

As shown in the heat map, the bacterial community of the three treatments was divided into two groups at first level (Fig. 3). One of them was composed of the CB group, and the other included the PC and NC groups, indicating that the bacterial community in the PC and NC treatments shared a high similarity and grouped into a branch apart from the CB group. **(**Fig. 3**).**

### LC-MS analysis

**Fig. 4:**
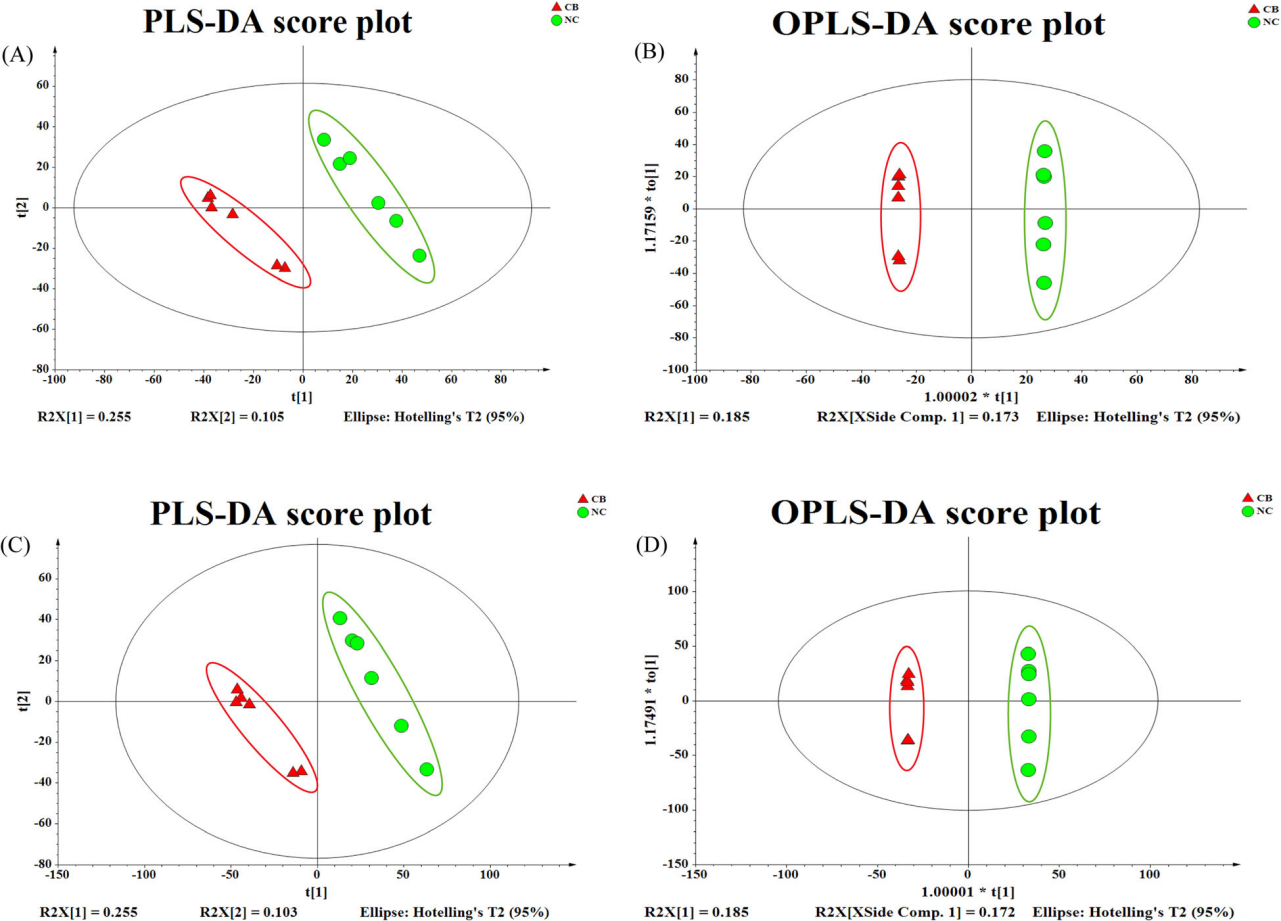
Partial least squares discriminant analysis (PLS-DA) and orthogonal partial least-squares discriminant analysis (OPLS-DA) two-dimensional score plots of fecal metabolites in comparisons of the NC and CB groups following (**a**), (**b**) positive and (**c**), (**d**) negative mode ionization

Figure 4a and c show the PLS-DA model that was used for dimensionality reduction analysis. Each point in the figure represents a sample and a PLS-DA model was obtained, with R2X [1]=0.255, R2X [2]=0.105 (positive mode ionization), and R2X [1]=0.255, R2X [2]=0.103 (negative mode ionization). To reduce the intra-group differences and further expand the differences between the groups, a supervised OPLS-DA analysis was used. As shown in Fig. 4b and d, the model parameters were R2X [1]=0.185, R2X[XSide Comp.1] = 0.173 (positive mode ionization), and R2X [1]=0.185, R2X[XSide Comp.1] = 0.172 (negative mode ionization). All the score plots of the faecal metabolites were carried out in the 95% Hotelling’s T2 ellipse and separated evidently. The results indicated that the model’s discrimination and prediction rate were good. The scattered point map constructed by the score plots showed that the two groups exhibited a certain separation, thereby proving that the differences in composition and concentration of the variables/molecules contained in the sample were greater. **(**Fig. 4**).**

### Metabolite content change

Student’s t-test was accustomed to test the difference of faecal metabolites in the control (NC) group and the *C. butyricum* (CB) group. A *P*-value< 0.05 and FC≧1.5 or FC≦0.667 was considered as the evidence of significant differences. It can be seen from Table 4 that 22 differential metabolites were achieved by qualitative comparison of mass spectrometry. Among them, 15 metabolites (citrulline, acetyl-DL-valine, L-citrulline, 4-hydroxy-L-proline, cimaterol, sebacic acid, suberic acid, azelaic acid, dodecanedioic acid, *o*-toluic acid, 3-methylglutaric acid, Indole-3-carboxylic acid, 4-acetamidobutanoic acid, nonic acid and 2-phenylpropionic acid) were enriched. Seven metabolites (L-valine, N-acetylhistamine, phosphatidylinositol lyso 16:0, phosphatidylethanolamine lyso 18:2, trans-3-coumaric acid, xanthurenic acid and 2,3-dihydroxybenzoic acid) were decreased in the pigs fed with *C. butyricum* diet compared with the control diet. Besides, nine metabolites belonged to organic acids including O-toluic acid, 3-methylglutaric acid, indole-3-carboxylic acid, 4-acetamidobutanoic acid, nonic acid, trans-3-coumaric acid, xanthurenic acid, 2,3-dihydroxybenzoic acid and 2-phenylpropionic acid. Five metabolites involved in amino acid were identified to be citrulline, acetyl-DL-valine, L-citrulline, L-valine and 4-hydroxy-L-proline. Six metabolites were classified into lipids, viz. phosphatidylinositol lyso 16:0, sebacic acid, suberic acid, azelaic acid, dodecanedioic acid and phosphatidylethanolamine lyso 18:2. Moreover, cimaterol and N-acetylhistamine were classified into drugs and an amine, respectively. **(**Table 4**).**

**Table 4:** Different endogenous metabolites in the feces of weaned piglets after adding *C. butyricum*

Super class	Metabolite names	Precurso type	m/z^a^	Rt^b^	log2fc_CB/NC^c^	*P*-value	Trend^d^
Amino acids	Citrulline	[M-H]-	174.09	101.75	1.71	< 0.001	↑
Amino acids	Acetyl-DL-Valine	[M-H]-	158.08	306.14	0.83	0.009	↑
Amino acids	L-citrulline	[M + H]+	176.10	102.20	1.05	0.027	↑
Amino acids	L-valine	[M-H]-	115.92	1010.69	−0.60	0.034	↓
Amino acids	4-hydroxy-L-proline	[M-H]-	129.98	207.29	0.80	0.044	↑
Drugs	Cimaterol	[M + H]+	220.15	565.94	0.95	0.012	↑
Amine	N-acetylhistamine	[M-H]-	152.08	778.56	−2.04	0.011	↓
Lipids	Phosphatidylinositol lyso 16:0	[M-H]-	571.29	738.01	−3.50	0.001	↓
Lipids	Sebacic acid	[M-H]-	201.11	561.47	1.42	0.001	↑
Lipids	Suberic acid	[M-H]-	173.08	452.26	1.19	0.011	↑
Lipids	Azelaic acid	[M-H]-	187.10	508.95	1.38	0.019	↑
Lipids	Dodecanedioic acid	[M-H]-	229.14	657.55	0.72	0.024	↑
Lipids	Phosphatidylethanolamine lyso 18:2	[M-H]-	476.28	827.81	−1.06	0.028	↓
Organic acids	O-toluic acid	[M-H]-	135.05	538.95	1.11	0.002	↑
Organic acids	3-methylglutaric acid	[M-H]-	145.05	380.25	1.53	0.003	↑
Organic acids	Indole-3-carboxylic acid	[M-H]-	160.04	504.67	1.38	0.014	↑
Organic acids	4-acetamidobutanoic acid	[M-H]-	144.07	264.59	1.23	0.015	↑
Organic acids	Nonic Acid	[M + H]+	189.11	513.04	1.72	0.016	↑
Organic acids	Trans-3-coumaric acid	[M-H]-	163.04	459.07	−1.48	0.023	↓
Organic acids	Xanthurenic acid	[M-H]-	204.03	464.26	−1.39	0.028	↓
Organic acids	2,3-dihydroxybenzoic acid	[M-H]-	153.02	397.38	−1.22	0.037	↓
Organic acids	2-phenylpropionic acid	[M-H]-	149.06	601.41	0.88	0.041	↑

^a^m/z, mass-to-charge ratio; ^b^RT, retention time; ^c^FC fold change; ^d^″↑/↓” indicate the increase/decrease in the metabolite level after adding *C. butyricum* to the diet (*n* = 6)

### Metabolic pathway analysis

**Fig. 5:**
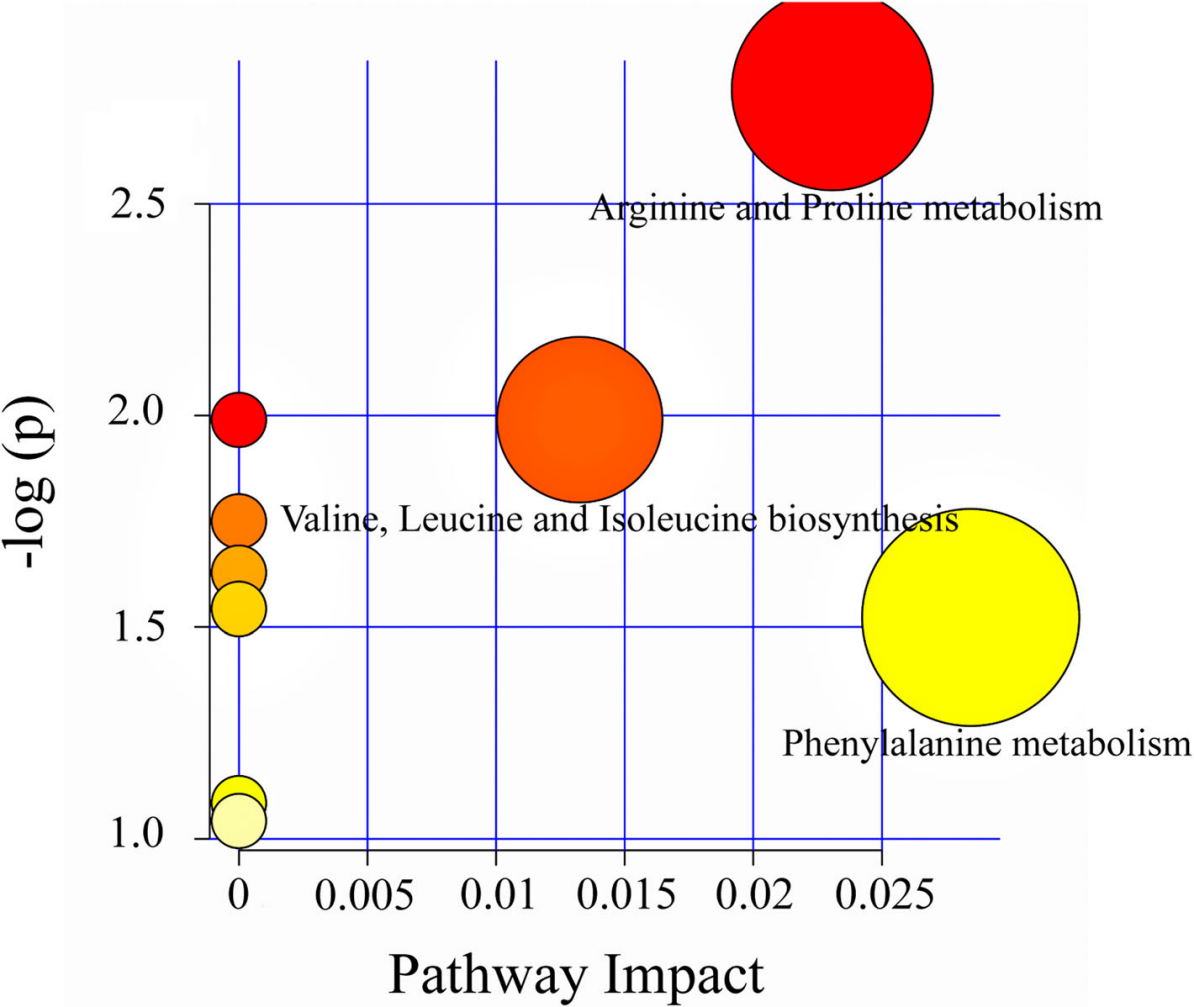
Pathway enrichment map analysis of differential metabolites in faeces between NC and CB groups using MetaboAnalystR 3.0. The colour of the circles from white to yellow to red denotes incremental fold change (−log(p)). The size of the circles from small to large indicates an increment of the impact of pathway

As shown in Fig. 5, pathway enrichment map analysis of differential metabolites in faeces between NC and CB groups using MetaboAnalystR 3.0. The differential metabolites between NC and CB groups were related to three pathways, including arginine and proline metabolism; valine, leucine and isoleucine biosynthesis; and phenylalanine metabolism. **(**Fig. 5**).**

### Realation in the growth performance, gut microbiota and metabolites

**Fig. 6:**
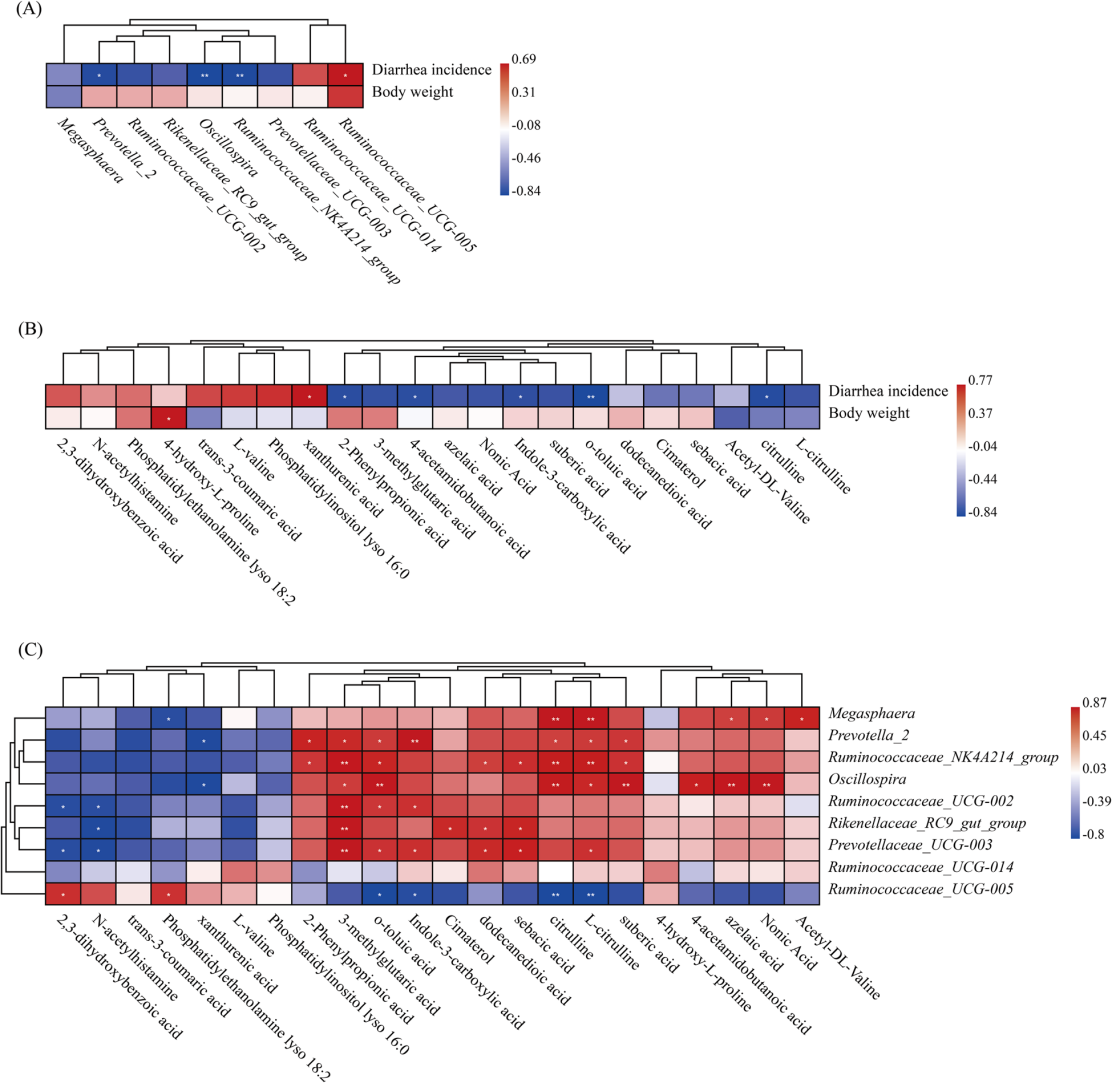
Correlation between the body weight, diarrhea incidence, differential microbiota (at the genera level) and metabolites. **a** Correlation between body weight, diarrhea incidence and microbiota. **b** Correlation between body weight, diarrhea incidence and metabolites. **c** Correlation between microbiota and metabolites. Strength (Spearman’s ρ value) and significance of correlations are shown as color in shades (red, positive correlation; blue, negative correlation). The values above/below zero represent positive/negative correlations. Significant correlations are noted by * *P* < 0.05, ** *P* < 0.01

The results of the Spearman correlation analysis are shown in Fig. 6. Different degrees of association was observed between the growth performance, the differential microbial genera and faecal metabolites as follows: diarrhea incidence was positively associated with *Ruminococcaceae*_UCG-005 (*P <* 0.05), while negatively associated with *Prevotella*_2, *Oscillospira* and *Ruminococcaceae*_NK4A214_group (*P <* 0.05) **(**Fig. 6a**)**. Diarrhea incidence was positively associated with xanthurenic acid (*P <* 0.05), while negatively associated with 2-phenylpropionic acid, 4-acetamidobutanoic acid, indole-3-carboxylic acid, *o*-toluic acid and citrulline (*P <* 0.05). Body weight was positively associated with 4-hydroxy-L-proline (*P <* 0.05) **(**Fig. 6b**).** *Megasphaera* was positively associated with citrulline, L-citrulline, azelaic acid, nonic acid and acetyl-DL-valine (*P <* 0.05), while negatively connected with phosphatidylethanolamine lyso 18:2 (*P <* 0.05). *Prevotella*_2 was positively associated with 2-phenylpropionic acid, 3-methylglutaric acid, *o*-toluic acid, indole-3-carboxylic acid, citrulline, L-citrulline, suberic acid (*P <* 0.05), while negatively associated with xanthurenic acid (*P <* 0.05). *Ruminococcaceae*_NK4A214_group was positively associated with 2-phenylpropionic acid, 3-methylglutaric acid, *o*-toluic acid, dodecanedioic acid, sebacic acid, citrulline, L-citrulline, suberic acid (*P <* 0.05). *Oscillospira* was positively associated with 3-methylglutaric acid, *o*-toluic acid, citrulline, L-citrulline, suberic acid, 4-acetamidobutanoic acid, azelaic acid, nonic acid (*P <* 0.05), while negatively associated with xanthurenic acid (*P <* 0.05). *Ruminococcaceae*_UCG-002 was positively associated with 3-methylglutaric acid, *o*-toluic acid, indole-3-carboxylic acid (*P <* 0.05), while negatively associated with 2,3-dihydroxybenzoic acid and N-acetylhistamine (*P <* 0.05). *Rikenellaceae*_RC9_gut_group was positively associated with 3-methylglutaric acid, cimaterol, dodecanedioic acid, sebacic acid (*P <* 0.05), while negatively associated with N-acetylhistamine (*P <* 0.05). *Prevotellaceae*_UCG-003 was positively associated with a number of metabolites, including 3-methylglutaric acid, *o*-toluic acid, indole-3-carboxylic acid, dodecanedioic acid, sebacic acid, L-citrulline (*P <* 0.05), while negatively connected with 2,3-dihydroxybenzoic acid and N-acetylhistamine (*P <* 0.05). *Ruminococcaceae*_UCG-005 was positively associated with 2,3-dihydroxybenzoic acid and phosphatidylethanolamine lyso 18:2 (*P <* 0.05), while negatively connected with *o*-toluic acid, indole-3-carboxylic acid, citrulline and L-citrulline (*P <* 0.05) **(**Fig. 6c**).**

## Discussion

Increasing evidences suggest the opinion that probiotics can enhance animal growth [43–45]. The application of *C. butyricum* as a feed additive in animals has been widely proven [14, 20, 46, 47]. However, there is a shortage of information on the microbial community and the metabolic profile, after the addition with *C. butyricum* in the feed in weaned piglets. In the current study, we investigated the microbiota diversity and metabolite profiles of weaned piglets fed *C. butyricum* using 16S rRNA MiSeq sequencing and LC-MS/MS. Our results showed that the intestinal microbial community and metabolic profiles were significantly different in piglets fed *C. butyricum* compared with control diet pigs.

Several reports have shown that *C. butyricum*-based probiotics exhibited a significant positive influence on growth performance in weaned piglets [13, 48]. It has been reported that *C. butyricum* addition to the diet led to an increase in the villus height and enlarged crypt depth, which points out that the absorptive capacity of the intestinal cells was raised and possibly contributing an improvement in the average daily gain. Another report pointed out that the reason for the reduction of diarrhea incidence was the production of butyric acid by *C. butyricum*. The production of butyrate in the colon inhibited the fatty acid synthesis, which resulted in the production of folic acid, and because of the role of folic acid in methylation and gene regulation, diarrhoea was reduced [49]. However, in this study, the addition of *C. butyricum* to the diet did not significantly affect the growth performance of the piglets. One unanticipated finding was that diarrhea incidence was significantly taller in the antibiotic addition group and *C. butyricum* addition group than that in the control group during 14–21 day feeding. A possible reason may depend on the amount of the antibiotics and *C. butyricum* additive, gut microbiota, environmental conditions and health status of the piglets, suggesting that a small stress was more likely to cause diarrhea in piglets treated with antibiotics and *C. butyricum*, before the stabilization of the intestinal microbial colonies.

As an imperative part of the intestinal tract, the intestinal microbiota is also known as the body’s black box. All life activities and metabolism, barrier function, nutrition, and immune response are closely related to human health [50]. The steady-state of the flora has an important influence on maintaining the body’s growth and development, nutrient digestion and absorption, and immune antagonism [51]. A richer microbiota composition readily reduces the incidence of disease and affects the health of the host [52]. Many researchers have reported that the guiding phyla in the gastrointestinal tract are Firmicutes and Bacteroidetes in pig fecal samples [44, 53, 54] and our results supported this. Firmicutes comprises a range of cellulolytic organisms, which are advantageous to cellulose decomposition [55]. Bacteroides can degrade high-molecular-weight organic matter and improve the innate immune response by enhancing the function of intestinal mucosal barrier [56, 57]. A huge number of investigations have shown that probiotic intervention can change the structure of intestinal flora by regulating the ratio of Firmicutes/Bacteroidetes [58] and Proteobacteria/Bacteroidetes [59]. Prior research indicated that there was a correlation between body weight and gut microbial ecology. The microbiota in obese subjects indicated an elevatory proportion of Firmicutes and a depressed population of *Bacteroides*. Likewise, an amplified Firmicutes/Bacteroidetes ratio has been directly linked to weight gain [60]. More studies have shown that the raise in Firmicutes with the decrease of Bacteroidetes in gut exert exhibited a strong connection with host lipid accumulation and fatty liver [20, 61, 62]. Our study identified that Bacteroidetes, Firmicutes and Tenericutes were found to be the dominant flora in the intestinal tract of piglets. Diets supplemented with *C. butyricum* showed a trend of increasing the ratio of Firmicutes/Bacteroidetes (*P* = 0.075). All this indicates that *C. butyricum* has the potential to promote the growth of piglets by increasing the Firmicutes/Bacteroidetes ratio.

*Prevotella* is the most abundant microorganism in the Bacteroidetes group, and it is an exceedingly active hemicellulose-decomposing bacterium that is essential for the degradation of plant non-fibrous polysaccharides and proteins [63, 64]. *Megasphaera elsdenii* is a major inhabitant of the pig intestine [65] and is a predominant and important bacteria that converts lactate to all sorts of short chain fatty acids (SCFAs), such as acetate, propionate, butyrate and valerate [54]. These SCFAs functions as an energy source for the host and plays a momentous role in gut health [66]. It has been reported that *Megasphaera* improved the gastrointestinal health of rats [67] and pigs [68] as a probiotic treatment. Some research indicated that *C. butyricum* enhanced the uniformity of the intestinal bacterial community and maintained a more balanced microbial structure in the weaned piglets. *Megasphaera* increased from 3.79 to 11.31% and became the main responder [54]. Similar to these consequences, the current researches presented that *C. butyricum* greatly improved the abundance of *Megasphaera.* Ruminococcaceae, a member of the Firmicutes, is one of the most abundant families in the order *Clostridiales*, which is associated with the maintenance of gut health [69]. It is reported that they primarily produce butyric acid that exerts probiotic physiological functions in the gut [70, 71]. Both *Ruminococcaceae*_UCG-002 and *Ruminococcaceae*_UCG-014 genera produce butyrate, which plays a dominant role in the colon health [70, 72]. Our investigations indicate that diet added with *C. butyricum* can enhance the relative proportion of *Megasphaera* and *Ruminococcaceae*_NK4A214_group, thereby enhancing the superior position of the Firmicutes in the intestinal flora. *Oscillospira* species are producers of butyrate, and at least some of them can utilize glucuronate [73]. A study shows that *Oscillospira* improved the metabolism [74] and had the ability to degrade host glycans [75]. In the present study, it was perceived that *C. butyricum* substantially. Increased the relative abundance of the genus *Oscillospira*. This suggests that *C. butyricum* can promote the metabolism by improving *Oscillospira* abundance. This indicates that *C. butyricum* can promote the metabolism by improving *Oscillospira* abundance. The results of correlation analysis illustrated that the abundance of *Prevotella*_2, *Oscillospira* and *Ruminococcaceae*_NK4A214_group were negatively correlated with the diarrhea incidence, *Ruminococcaceae*_UCG-00*5* was positively associated with diarrhea incidence, indicating that the relative abundance of *Prevotella*_2*, Oscillospira*, *Ruminococcaceae*_NK4A214_group and *Ruminococcaceae*_UCG-00*5* were important intestinal fecal bacteria, which closely related to the diarrhea.

Metabonomics has been labelled as one of the new ‘-omics’, joining genomics, transcriptomics and proteomics as a science employed for the understanding of global systems biology [76]. Metabonomics has been widely applied in many research areas, including drug toxicology [77], biomarker discovery [78], functional genomics [79], molecular pathology [80] and animal nutrition [81]. Similarly to other omics, the subtle changes in metabolite content are directly related to large changes in biological phenotype [82]. Previous research has shown that *C. butyricum* might be a practicable probiotic to decrease the saturated fatty acids contents and increase the monounsaturated fatty acids and polyunsaturated fatty acids contents of Peking duck meat [83].

Citrulline is a common intermediate metabolic molecule in mammals. It is an amino acid that is not involved in protein synthesis, but is closely related to arginine metabolism [84]. In the current study, the levels of citrulline and L- citrulline in the metabolites were significantly increased in the faeces of the piglets with *C. butyricum* supplemented diet. One interesting finding was that the content of citrulline in faecal metabolites was positively associated with the abundance of *Megasphaera*, *Prevotella*_2, *Ruminococcaceae*_NK4A214_group, *Oscillospira*, while negatively correlated with the diarrhea incidence and *Ruminococcaceae*_UCG-005 in faecal microorganisms. Comprehensive analysis of bacteria in the genus, we conclude that *C. butyricum* mainly increases the content of citrulline in faecal metabolites by increasing *Megasphaera*, *Ruminococcaceae*_NK4A214_group and decreasing the abundance of *Ruminococcaceae*_UCG-005.

Valine is one of the most important essential amino acid in pigs, and belongs to branched-chain amino acids [85]. It is a glycemic amino acid [86]. After transamination, oxidative decarboxylation and dehydrogenation, succinic monoacyl CoA is produced, which enters into the tricarboxylic acid cycle and supplies energy to the body. In this study, the amount of L-valine was decreased and acetyl-DL-valine was increased by *C. butyricum* treatment, which may contribute to the disturbance of valine metabolism. 3-Methylglutaric acid is a metabolite derived from leucine catabolism [87]. In our research, an increase in the level of 3-methylglutaric acid indicates the changes in leucine metabolism.

The significance of proline has been extensively exhibited to act a pivotal part in cell structure, anti-oxidative reactions, immune responses, energy metabolism and protein synthesis in more plentiful organisms [88]. The addition of proline to the standard abalone diets has been found to act as a substrate for the amino acid catabolism in slower-growing abalone, to utilize the decomposition of proline to aid in the generation of energy through the tricarboxylic acid cycle [89]. In our study, dietary *C. butyricum* increased the levels of 4-hydroxy-L-proline and 4-hydroxy-L-proline was positively correlated with body weight, suggesting that the proline metabolism pathway was disturbed.

Cimaterol is a kind of beta doping. In animal husbandry production, a large dose of cimaterol can reduce the fat content of the carcass, improve the proportion of lean meat, promote the growth of livestock and achieve the effect of improving meat quality [90, 91]. Histamine plays a very significant role in the various physiological function of immune, nervous and gastrointestinal systems. There have been reports that histamine is associated with secretion of gastric acid in the gastrointestinal system [92]. In our study, dietary *C. butyricum* decreased the levels of the N-acetylhistamine and the content of N-acetylhistamine was negatively correlated with the abundance of *Ruminococcaceae*_UCG-002, *Rikenellaceae*_RC9_gut_group, and *Prevotellaceae*_UCG-003. This finding suggests that *C. butyricum* could cause the abnormal histidine metabolism, alongwith an abundance of beneficial bacteria in the gut.

Suberic acid is a colorless crystalline dibasic acid. It has been reported that a great potential exists for suberic acid to be developed as an anti-photoaging agent [93]. Azelaic acid is a natural saturated dicarboxylic acid that is useful for the treatment of comedonal acne and inflammatory acne [94]. Dodecanedioic acid is a dicarboxylic acid with 12 carbon atoms. It has not been confirmed for intestinal absorption, but has been indicated hepatic and renal uptake [27]. In the present study, medium-chain dicarboxylic acids (suberic and azelaic acid) and long-chain dicarboxylic acids (sebacic acids, dodecanedioic acid) were improved in the *C. butyricum* addition group as compared with the control group. This showed that *C. butyricum* enhances the fatty acid oxidation in piglets. Moreover, sebacic acids and dodecanedioic acid were found to be associated positively with *Ruminococcaceae*_NK4A214_group, *Rikenellaceae*_RC9_gut_group and *Prevotellaceae*_UCG-003. Suberic was associated positively with *Prevotella*_2, *Ruminococcaceae*_NK4A214_group, *Oscillospira*. Azelaic acid was associated positively with *Megasphaera* and *Oscillospira*. In this pursuit, we suggest that the gut microbiota has a large metabolic potential and can impact the host’s nutrition significantly. Recent studies have further reinforced that gut microbiota affects host health and disease [27].

Indole-3-acetic acid is a metabolite of tryptophan, which is mainly produced by direct or indirect metabolism of the intestinal microbiota [95]. About 4–6% of tryptophan is degraded by bacteria to produce indole metabolites [96]. Other studies have suggested that indole, a microbial breakdown product of tryptophan, regulate the integrity of intestinal tight junctions [97]. It was proposed that indol metabolites and their gut bacterial producers play an important role in overweight related inflammation in young adults [98]. Our results showed that indole-3-carboxylic acid was upregulated and the content of indole-3-carboxylic acid was positively correlated with the abundance of *Prevotella*_2, *Ruminococcaceae*_UCG-002, *Prevotellaceae*_UCG-003, while negatively correlated with diarrhea incidence and *Ruminococcaceae*_UCG-005 abundance, suggesting that *C. butyricum* can affect the growth health of piglets by regulating the indolic tryptophan metabolic pathway. Xanthine acid is one of several metabolites involved in the kynurenine pathway of tryptophan metabolism [99], which promotes increased bone density and is associated with a lower risk of fracture [100]. Based on our results, it appeared that the levels of xanthurenic acid were downregulated and the content of xanthurenic acid was positively correlated with diarrhea incidence, while negatively associated with the abundance of *Prevotella*_2 and *Oscillospira*. An earlier study has demonstrated that there was reciprocal relationship between gut microbes and several bacterial metabolites that facilitates changes in intestinal homeostasis [101].

## Conclusions

In the present study, we examined the overall comprehension of the patterns of microbial colonization and metabolite composition in healthy piglets using *C. butyricum* as a feed additive. Our results demonstrate that the shift of the fecal microbiome composition and concentration, and the colonization of potential probiotics was accelerated by *C. butyricum*, which may modulate the host metabolism and enhance the intestinal development. Furthermore, Spearman’s analysis revealed an obvious correlation between the microbiota and the metabolites, indicating that *C. butyricum* presented the potential protective impact through gut microbiota and other pathways.
